# Design and Engineering of a Palm-Sized Optical Immunosensing Device for the Detection of a Kidney Dysfunction Biomarker

**DOI:** 10.3390/bios12121118

**Published:** 2022-12-02

**Authors:** Divya, Supratim Mahapatra, Pranjal Chandra

**Affiliations:** Laboratory of Bio-Physio Sensors and Nanobioengineering, School of Biochemical Engineering, Indian Institute of Technology (BHU) Varanasi, Varanasi 221005, Uttar Pradesh, India

**Keywords:** optical sensing device, paper sensor, personalized diagnosis, surface chemistry, kidney dysfunction

## Abstract

Creatinine is one of the most common and specific biomarkers for renal diseases, usually found in the serum and urine of humans. Its level is extremely important and critical to know, not only in the case of renal diseases, but also for various other pathological conditions. Hence, detecting creatinine in clinically relevant ranges in a simplistic and personalized manner is interesting and important. In this direction, an optical sensing device has been developed for the simple, point-of-care detection of creatinine. The developed biosensor was able to detect creatinine quantitatively based on optical signals measured through a change in color. The sensor has been integrated with a smartphone to develop a palm-sized device for creatinine analysis in personalized settings. The sensor has been developed following facile chemical modification steps to anchor the creatinine-selective antibody to generate a sensing probe. The fabricated sensor has been thoroughly characterized by FTIR, AFM, and controlled optical analyses. The quantitative analysis is mediated through the reaction between picric acid and creatinine which was detected by the antibody-functionalized sensor probe. The differences in color intensity and creatinine concentrations show an excellent dose-dependent correlation in two different dynamic ranges from 5 to 20 μM and 35 to 400 μM, with a detection limit of 15.37 (±0.79) nM. Several interfering molecules, such as albumin, glucose, ascorbic acid, citric acid, glycine, uric acid, Na^+^, K^+^, and Cl^−^, were tested using the biosensor, in which no cross-reactivity was observed. The utility of the developed system to quantify creatinine in spiked serum samples was validated and the obtained percentage recoveries were found within the range of 89.71–97.30%. The fabricated biosensor was found to be highly reproducible and stable, and it retains its original signal for up to 28 days.

## 1. Introduction

Chronic kidney disease has emerged as one of the most common conditions affecting more than 10% of the world’s population, which equates to more than 800 million people. Over the last two decades, an exponential surge, i.e., a 42% increase in the number of deaths caused by chronic kidney disease was recorded worldwide [1]. According to the Global Burden of Disease Study, there has been a surge in mortalities due to chronic kidney disease and it has become one of the major diseases, leading to a global burden [2,3]. There are various risk factors associated with chronic kidney disease and co-morbid conditions which make the condition worse for patients. Chronic kidney disease was found to more prevalent in cardiovascular patients [4]. In a recent study, it was shown that cases of chronic kidney disease have become common in children and thus the global burden has increased and is hampering children’s quality of life. In most pediatric cases, it was observed that children require dialysis or kidney transplants which are not easily available in developing and under-developed countries [5,6]. In another study, it was established that depression and anxiety also contribute to the suffering of people with chronic kidney disease [7]. The divergence in the prevalence and severity of chronic kidney disease is possibly due to the complex interaction between biological and non-biological risk factors. Therefore, diagnosing chronic kidney disease becomes tedious and important, especially targeting more specific biomarkers. In this critical condition, the kidneys are not able to filter out the waste of natural atrophies from the body [8]. The kidneys’ filtration ability can be calculated by finding the glomerular filtration rate (GFR), which also depends on a person’s age, sex, race, and weight. A decrease in value of the GFR represents a kidney problem and on the basis of the GFR value, kidney damage has been categorized in five different classes. Stage 1, normal kidney functioning, has a GFR value of more than 90 and Stage 5, kidney failure, has a GFR value of less than 15 [9,10]. Usually, the kidney filters out the various nitrogen-based waste materials and metals, including urea, uric acid, creatinine, ammonium, and lead. Numerous biomarkers and bioanalytical tests for diagnosing renal diseases include: the albuminuria, serum creatinine, creatinine clearance, glomerular filtration rate, and albumin-to-creatinine ratio [11,12]. Most renal infections are diagnosed based on the presence of various biomarkers in extracellular fluids [13]; however most of them are also related to several other pathological conditions [14]. Some bioanalytical tests, especially the glomerular filtration rate, are affected by age, gender, and lifestyle [15]. Therefore, selecting biomarkers is also one of the major criterion for properly diagnosing kidney disease. Serum creatinine is a more specific biomarker preferred globally by medical practitioners for kidney damage [16]. Creatinine is a metabolic product of creatine phosphate produced by a nonenzymatic reaction and its excretion is the major indication of the state of the kidney’s function [17]. The creatinine released in human blood and urine is critically important to the kidney, thyroid, and muscular functions [18,19]. The creatinine concentration in serum is not influenced by protein intake, so the level of creatinine in serum acts as a more suitable and effective indicator of kidney health. The normal physiological level of creatinine in blood serum lies in the range of 45–140 μM, and it exceeds this in different diseases/conditions [20]. The routine laboratory test for chronic kidney patients is mostly a urine analysis, indicating the presence of albumin/protein in the urine. Even the amount of microalbumin can indicate a serious renal infection but it is also associated with urinary tract infections. Additionally, in urine analyses, urinary sediments, such as hyaline casts, specific gravity, the color of the urine, and/or muddy brown casts are considered equally important [21,22]. Apart from urine, blood urea nitrogen and creatinine concentration tests are also recommended, which require many hours for testing and thus are not feasible for rapid, on-site detection. Many researchers have attempted to develop sensors based on various transduction systems for the detection of creatinine using various methods, i.e., optical [23,24], electrochemical [25,26], and chromatographic techniques [27,28]. Apart from the optical system, other methods require expensive and dedicated instruments and skilled personnel to perform the analysis, so they are not very handy for routine analyses [29,30]. In addition, optical sensors show an edge, especially in the medical field, because they can provide vital and complementary information without requiring laborious efforts. The most common optical methods for creatinine detection are based on Jaffe’s reaction [31] or an enzymatic reaction [32]. The optical sensors relying this reaction are majorly performed in the solution phase and do not utilize bioreceptors, such as antibodies or aptamers [31]. Moreover, analyses in the solution phase may also be compromised when the target is present in real samples, i.e., serum, urine, or other extracellular fluids [33]. The problem with such optically developed systems is mainly related to selectivity. Hence, the engineering of a specific bioreceptor onto a solid surface can be utilized to develop a selective sensor system. Among various surfaces, a paper-based matrix is the most simplistic one and has been greatly utilized to fabricate various types of biosensors [34,35,36].

Thus, we attempted to incorporate the anti-CR antibody onto a paper surface to make the reaction more selective and sensitive. Although a few attempts were made to develop a lateral flow assay-based kit for creatinine detection, especially for kidney patients, in such a system, there is a chance of an interruption in the flow of the target analyte due to the profusion of pores [34,37]. This issue has been examined and resolved by using a paper disc, which does not require any sample movement, thus having an edge on other systems [34,38]. Paper-based systems are more commercially viable, easy to use, and environmentally suitable. Conventionally, optical signatures are subjected for red/green/blue (RGB) analyses, which depend on the changing intensities of these colors. These changes in color intensities help to obtain the calibration plot for analyte detection associated with diverse biomolecular and chemical reactions [39,40]. Hence, the relationship between such changes in color and the analyte concentration needs to be established for the quantitative detection of creatinine.

In this work, we have developed a hand-held, on-site, miniaturized, point-of-care diagnostic device for the sensitive and quantitative detection of creatinine in serum samples in regards to renal disease. A cellulosic filter paper was selected as a probe material on which the sensor surface was constructed. The sensor surface was fabricated through chemical modifications and antibody immobilization for specific and sensitive creatinine measurements. Further, the fabricated probe was thoroughly characterized using Fourier transform infrared spectroscopy (FTIR), atomic force microscopy (AFM), and controlled colorimetric studies after each step, and assembled on a paper strip. To the best of our knowledge, this is a first report in which a paper-based integrated immunosensing device has been developed for the direct detection of creatinine. After that, we designed a miniaturized hardcase-bound prototype of a device in which a dedicated imaging tunnel was constructed. The device is user-friendly and allows a user to capture the probe’s image using a smartphone. The color condition and the color-changing phenomena indicating the proportional change in the analyte concentrations were detected through the developed device. The selectivity, real sample analysis, and reproducibility were also validated to establish the suitability of the overall sensing device’s market potential and application in daily life.

## 2. Experimental Section

### 2.1. Materials and Reagents

All the chemical reagents were purchased from standard companies and further used without any purification steps. Deionized water was used to prepare the buffer solutions for the experiments following basic laboratory methods. Creatinine (Cr), sodium nitrite, o-nitrobenzoic acid, picric acid (PA), 1-(3 dimethylaminopropyl) 3-ethyl carbodiimide hydrochloride (EDC), n-hydroxysuccinimide (NHS), Na_2_HPO_4_, NaH_2_PO_4_, sodium chloride, serum albumin, lactose, citric acid, uric acid, and potassium chloride were procured from SRL India. Grade-1 Whatman filter paper was purchased from Whatman Inc., Cytiva, MA, USA. Standard serum (RM 9955) and Tween-20 was procured from Hi-media, Maharashtra, India. Anti-CR antibody (ab30719) was procured from Abcam, Inc., Cambridge, UK. EDC/NHS was prepared in 0.1 M PBS (pH -5). Volumes of 0.01 M PBS (pH 7.4) and 0.01 M PBS-Tween (pH 7.4; 1% tween) buffer were prepared using the standard method [41]. Diazonium salt (0.05 M) was prepared by adding 184.5 mg o-nitrobenzoic acid, 130.0 mg sodium nitrite, and 36.0 mg ascorbic acid sequentially in 45.0 mL HCl (1 M).

### 2.2. Designing of Sensing Probe

**Figure 1:**
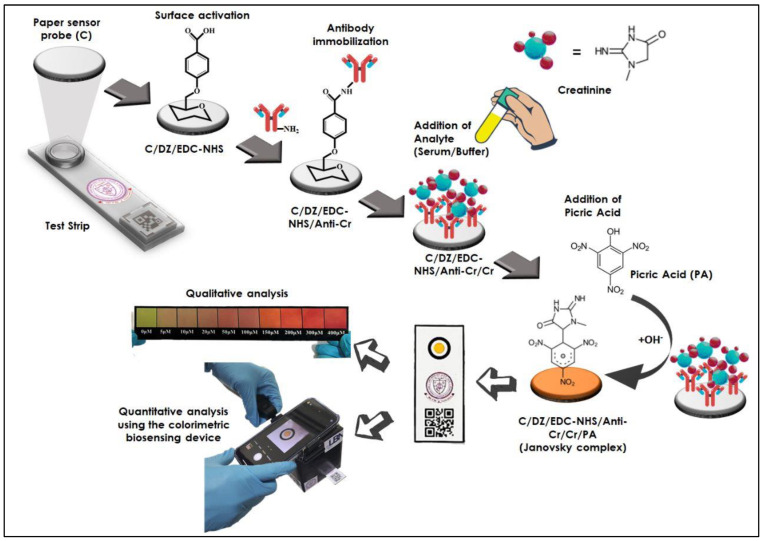
Illustration of the step-by-step fabrication of the sensor probe and the proposed colorimetric sensing device.

Grade 1 Whatman filter paper (C) was selected as the base material for the fabrication of the sensor probe. For this, a Whatman filter paper was cut into circular discs using a paper-punching machine to have a defined diameter of 5.0 mm for all discs. After that, the discs were treated with 4-carboxybenzene diazonium (DZ) salt by soaking them for at least six hours. Then the discs were thoroughly washed using a sterile PBS solution forming the C/DZ surface. The disc’s surface (C/DZ) was further treated with 5.0 µL EDC/NHS (140–100 mM, freshly prepared), which helps activate the carboxyl groups on the C/DZ surface, forming the C/DZ/EDC-NHS surface. After washing, 2.0 µL anti-CR (1.7 µg/µL) antibody was immobilized over the C/DZ/EDC-NHS surface. The immobilization occurred because of the reaction between the amino groups of the anti-CR antibody and carboxyl groups on the modified C/DZ/EDC-NHS surface. After this step, the developed probe was presented as C/DZ/EDC-NHS/anti-CR. Finally, to avoid false-positive signal generation and minimize nonspecific interaction, the surface (C/DZ/EDC-NHS/anti-CR) was treated with 1% BSA for 30.0 min. This step was further completed by washing the probe with autoclaved PBS-Tween for about 1.0 min to remove unabsorbed BSA. The fabrication step of each layer was confirmed by different techniques, i.e., FTIR and AFM, and optical techniques. Figure 1 illustrates the detailed step-by-step sensor probe fabrication method and the proposed colorimetric sensing device.

### 2.3. C/DZ/EDC-NHS/Anti-CR Biosensor Probe Testing

The creatinine was determined through the reaction between the final sensing probe C/DZ/EDC-NHS/anti-CR and creatinine, forming C/DZ/EDC-NHS/anti-CR/Cr. To test the ability of the designed probe, different concentrations of creatinine (3 µL) were utilized and allowed to react with 2 µL of PA (1%) for 7.0 min (optimized). It was found that this reaction produced a reddish-orange-colored complex catalyzed by creatinine immunocomplexed to the C/DZ/EDC-NHS/anti-CR probe. The reaction between creatinine and picric acid was also shown in the illustration (Figure 1). After that, the probe was assembled on a paper strip and fitted to the device prototype. A smartphone camera (iPhone 13, dual 12-megapixel camera) was used to record the color change in the probe in particular conditions, followed by image processing. The quantification of color change was performed by processing the corresponding RGB values of a specific color. The image processing was performed in such a manner that the colored probe area was specifically selected for analysis, so that the error in RGB values and false-positive results can be avoided during the imaging process. This area-selection method also helps determine the accurate color intensity change by calculating the mean intensity of color in the uniformly distributed area.

The effective RGB intensities of the paper disc with color change were determined by using Equation (1):

(1) $$Effective\;I_{RGB}=\log_{10}\frac{I_{RGB}\left(Blank\right)}{I_{RGB}\left(Conc.\right)}$$

where I_RGB_ represents the mean intensities of all primary color channels obtained from the selected area of PA-treated C/DZ/EDC-NHS/anti-CR/Cr probe surface.

## 3. Experimental Results

### 3.1. Color Channel Selection

**Figure 2:**
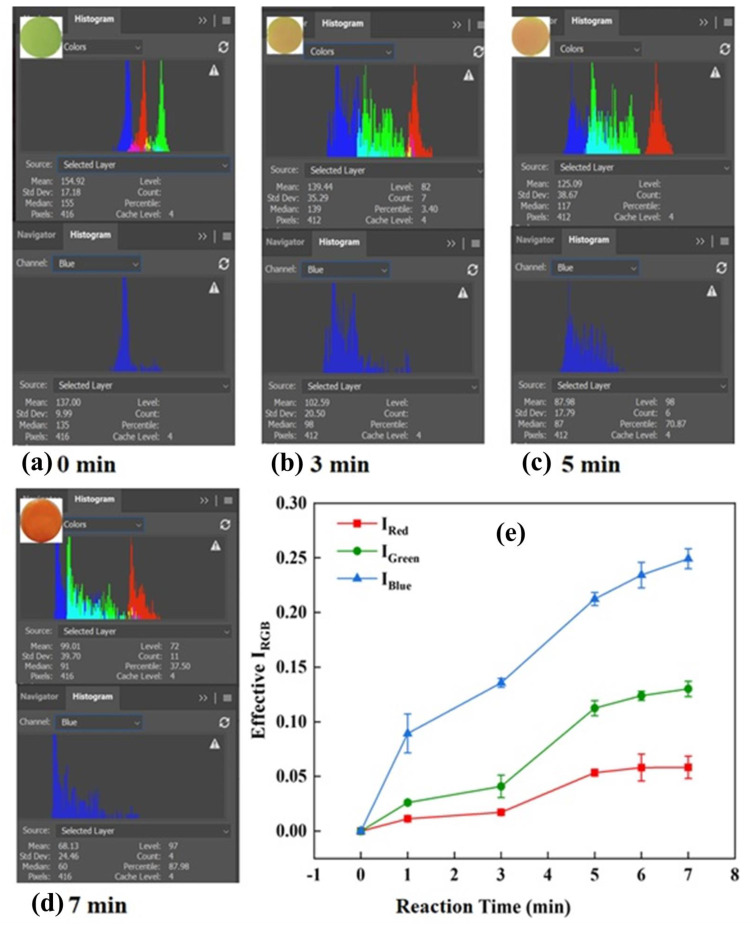
(**a**–**d**) Histogram showing the change in color intensity along with the real image of disc showing real–time color change; and (**e**) Representation of change in color intensity of all the three standards through RGB analysis, i.e., Effective I_Red,_ Effective I_Green,_ and Effective I_Blue_; symbols and bars represent the average and standard errors of the data (*n* = 3), respectively.

The image analysis was performed by following the RGB convention to assess the biosensing studies, in which, initially, the most efficient and sensitive primary color channel was chosen and examined. The data present in the digital image analysis were determined in the form of effective I_RGB_, which reflects the true pixel intensity of the color (a reddish-orange color in this reaction). I_RGB_ consists of the intensities of three different principal color passages, which are represented as I_Red_, I_Green_, and I_Blue_. The optical change shows the gradual change in the intensity of the color appearing and represents the complementary color absorption. Finally, to evaluate the analytical performance of the developed device depending upon the maximal change in sensitivity for the primary color channel, we performed a comparative analysis of all three primary color channels individually. Figure 2a–d represents the change in color to reddish-orange in a time-dependent manner on the developed sensing probe and the complementary histograms in RGB and B at 0.0, 3.0, 5.0, and 7.0 min. After that, we focused on calculating the individual Effective I_Red,_ Effective I_Green,_ and Effective I_Blue_ in a time-dependent manner from 1.0 to 7.0 min. The obtained data were plotted with respect to the changes in time as shown in Figure 2e. It was interesting to note that the highest signal change was observed for Effective I_Blue_ (0.1138 (±0.0060) a.u./min)_,_ in comparison to Effective I_Green_ (0.0619 (±0.0024) a.u./min) and Effective I_Red_ (0.0248 (±0.0039) a.u./min), which was also statistically significant (*p <* 0.003; *n* = 3). Therefore, in all further sensing analyses, Effective I_Blue_ was considered as an analytical parameter calculated using Equation (2):

(2) $$Effective\;I_{Blue}=\log_{10}\frac{I_{Blue}\left(Blank\right)}{I_{Blue}\left(Conc.\right)}$$

where I_Blue_ represents the mean pixel intensity of the blue channel determined from the sensing probe C/DZ/EDC-NHS/anti-CR/Cr discs when treated with picric acid.

### 3.2. Characterization of C/DZ/EDC-NHS/Anti-CR/Cr Probe

**Figure 3:**
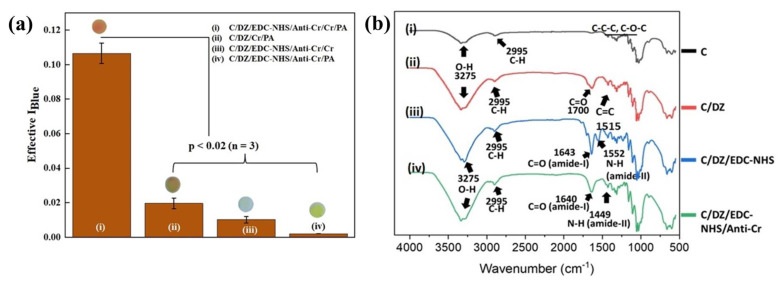
(**a**) Histograms showing the comparative Effective I_Blue_ of the different sensing probes: (i) C/DZ/EDC–NHS/anti–CR/Cr/PA (ii) C/DZ/Cr/PA (iii) C/DZ/EDC–NHS/anti–CR/Cr (iv) C/DZ/EDC–NHS/anti–CR/PA; (**b**) FTIR spectra of different stages of probe: (i) C, (ii) C/DZ, (iii) C/DZ/EDC–NHS, and (iv) C/DZ/EDC–NHS/anti–CR.

The developed sensing probe and the surface reactions were characterized step-by-step by various techniques, which include an optical analysis, FTIR, and AFM. An experiment showing the immunocomplexation formation was designed and performed to authenticate all the fabrication steps of the sensor probe. This study allowed the creatinine to react with the final sensing probe, i.e., C/DZ/EDC-NHS/anti-CR/Cr, which was further washed and finally exposed with PA to form the reddish-orange color, which is represented in Figure 3(ai). We have characterized the different sensing probe surfaces to confirm and validate that the color change intensity was simply because of the immunocomplex formation in addition to the catalytic reaction between creatinine and PA by conducting several control experiments. Such controlled experiments are extremely important to verify that the signals are not false-positive signals, but they are generated merely due to the antigen–antibody reaction. In the first controlled experiment, creatinine was allowed to react with the Diazonium-activated paper disc (C/DZ), which further interacted with the PA, developing the C/DZ/Cr/PA surface (Figure 3(aii)). In this case the obtained Effective I_Blue_ value was found to be 0.024 (± 0.003), which was approximately five times lower compared to the final probe (Figure 3(ai)). In the next controlled experiment, the creatinine conjugated surface was examined and was not treated with PA (C/DZ/EDC-NHS/Anti-CR/Cr) (Figure 3(aiii)). After the examination, the obtained Effective I_Blue_ value was found to be 0.018 (±0.001), which was approximately 8.1 times lower compared to the final probe (Figure 3(ai)). In contrast, in another controlled study, creatinine was not added to the sensing probe, but the probe was allowed to react with PA (C/DZ/EDC-NHS/Anti-CR/PA) (Figure 3(aiv)). In this case the Effective I_Blue_ value was found to be 0.0022 (±0.0001), which was approximately 53.2 times lower compared to the final probe (Figure 3(ai)). From the obtained controlled data, negligible signals were obtained, and hence, the fabricated sensor did not generate any false-positive results. These results were statistically evaluated by a *t*-test in which the *p*-value was obtained to be <0.02; *n* = 3.

Further, the design of the sensor probe was also confirmed through a layer-by-layer characterization of the probe by FTIR. The FTIR spectra for the untreated cellulose paper (C) disc, the cellulose paper surface treated with diazonium substrate (C/DZ), the diazonium-activated paper surface with EDC-NHS (C/DZ/EDC-NHS), and the antibody-immobilized final sensing surface (C/DZ/EDC-NHS/anti-CR) are shown in Figure 3b. Representative peaks appeared between 1000 and 1200 cm^−1^, 2995 cm^−1^, and 3275 cm^−1^ because of the vibrations of C-C-C and C-O-C, and the stretching vibrations of C-H and O-H, respectively, in the case of C (Figure 3(bi)). After this, the discs were treated with the diazonium solution and the peaks are represented in Figure 3(bii). Here the peaks appear at 1700 cm^−1^ and 1515 cm^−1^, which complements the C=O group and C=C group, respectively. These peaks are probably because of the introduction of carboxybenzyl flanking groups on the surface of the cellulose paper that lead to the formation of the C/DZ surface. In the third step, the discs were allowed to react with EDC/NHS over the modified C/DZ surface in which two sharp peaks of amide I and amide II at 1643 cm^−1^ and 1552 cm^−1^ were observed, respectively (Figure 3(biii)). These peaks indicate that the bending vibrations of amide bonds formed when they were allowed to be coupled with NHS and carboxybenzyl flanking groups on the surface. These EDC/NHS-modified cellulose surface peaks in the FTIR spectra have also been recorded previously [35]. Finally, in the last step, the anti-CR was immobilized on the paper surface to form the final sensing probe, i.e., C/DZ/EDC-NHS/anti-CR, which was confirmed by the presence of two specific bands of amide I and amide II, visible in Figure 3(biv). However, after the modification with anti-CR, the amide I and II peaks slightly shifted from their position as found on the EDC/NHS-modified surface [42]. These observed stretching vibrations are the direct indications of the backbone conformation of the protein (anti-CR), which indeed demonstrates the presence of immobilized anti-CR on the paper surface. Each layer was also characterized with reference to the thickness and surface topology using AFM.

**Figure 4:**
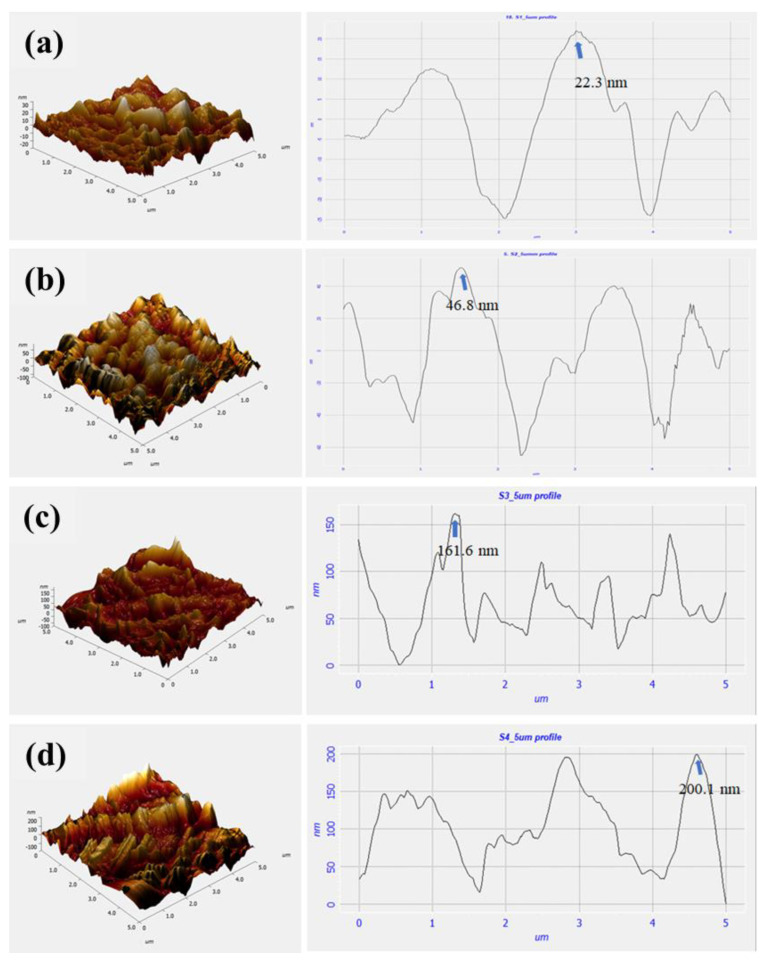
AFM images (**a**) C (z–deflection—22.3 nm), (**b**) C/DZ (z–deflection—46.8 nm), (**c**) C/DZ/EDC–NHS (z–deflection—161.6 nm), and (**d**) C/DZ/EDC–NHS/anti–CR (z–deflection—200.1 nm) representing the micrograph and corresponding z–deflection.

AFM was performed to validate the sensor-probe fabrication, in which the morphologies of every modified surface have been recorded in the contact mode (Figure 4). Firstly, the initial surface morphology of the bare cellulose paper (C) was obtained, in which a comparatively smoother topology of the fibrous paper surface appeared with a z-deflection value of 22.3 nm (Figure 4a). After which, on the second surface, which was formed by treating the paper surface with 4-carboxybenzene diazonium salt (C/DZ), significant granularity was observed with an increased z-deflection of 46.8 nm, due to the conjugation of carboxybenzyl molecules on the surface of the cellulose (Figure 4b). In the later stage, when the surface was treated with EDC-NHS to form the C/DZ/EDC-NHS surface, a more significant increase in the z-deflection value of 161.6 nm was recorded (Figure 4c). In the final step of the fabrication, upon immobilizing the anti-CR, the difference in the surface topology was changed with the increased z-deflection value of 200.1 nm (Figure 4d), which was merely due to the immobilization of anti-CR on the C/DZ/EDC-NHS surface. The results from all three characterization techniques, the image analysis (RGB), FTIR, and AFM, complement each other. Therefore, we concluded the layer-by-layer synthesis of the sensing probe was successful.

### 3.3. Analytical Performance of C/DZ/EDC-NHS/Anti-CR Biosensor

**Figure 5:**
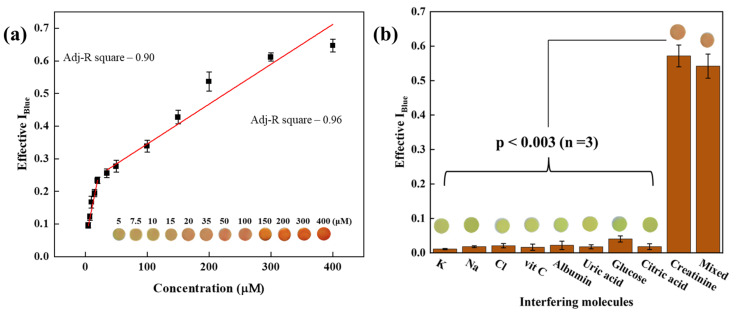
(**a**) Standard calibration graph showing two different dynamic ranges of creatinine from 5 to 20 μM and from 35 to 400 μM; (**b**) Selectivity assay of C/DZ/EDC-NHS/anti-CR sensor probe towards interfering molecules present in the serum and mixed-sample analysis.

After performing the characterization of the surface of the fabricated sensor probe, its analytical performance needed to be tested and evaluated by measuring the different concentrations of creatinine. To achieve this, initially, the developed sensing probe was allowed to react with the buffer only in the absence of creatinine, followed by the addition of PA, in which no significant Effective I_Blue_ was observed. After that, the developed probe C/DZ/EDC-NHS/anti-CR was tested against the different concentrations of creatinine followed by the reaction with PA, and the change in color was captured using the developed sensing device. Figure 5a displays the Effective I_Blue_ responses of the developed biosensing device in the absence and presence of creatinine in varying concentrations between 5 and 400 μM. The color change on the probe surface indicates the increase in the Effective I_Blue_ values with the increasing concentrations of creatinine. This proportional change in the effective I_Blue_ values with the change in creatinine concentration has shown that the designed sensing system can effectively and efficiently detect creatinine. Two different calibration plots with different analytical parameters have been obtained, based on the response of the Effective I_Blue_ values. The regression equations for creatinine are represented for low (5–20 µM) and high (35–400 µM) concentrations and the calibration plots are as shown: Effective I_Blue_ (a.u.) = 0.049 (±0.008) + 0.009 (±0.007) [Cr(µM)] and Effective I_Blue_ (a.u.) =  0.243 (±0.019) + 0.001 (±0.009) [Cr(µM)] with adjacent R^2^ values of 0.90 and 0.96, respectively. The detection limit of creatinine was determined by considering the calibration curve of the lower concentration (5–20 µM) to be 15.37 (±0.79) nM based on the standard deviation obtained from repeated readings of the blank with a 95.0% confidence level, *n* = 3. The LOQ was calculated using the lower calibration curve and found to be 50.72 nM (±2.14). The obtained detection limit is lower [43] or comparable [44] to those of the recently reported optical creatinine sensors. The analytical performance of various sensors in terms of the detection time, real sample, detection range, and limit of detection, including sensor configuration, is comprehensively described in Table 1.

It is worth mentioning that the developed system was able to measure the amount of creatinine that includes its clinical range in human serum and urine [20,45]; hence, it can be utilized for routine clinical tests in diverse matrices. Interestingly, the detection time of the developed device was recorded to be 7.0 min which is relatively faster [45] or comparable [47] to that of the previously reported method.

### 3.4. Selectivity Assay

To estimate the medical applicability of the designed system, the selectivity needs to be tested as it is one of the most critical parameters. Therefore, a selectivity assay was performed with the molecules that are majorly present in the serum matrix, i.e., glucose, bovine serum albumin (BSA), uric acid, citric acid, vitamin C, and commonly present ions (Na^+^, K^+^, Cl^−^) separately, under the same experimental conditions. Figure 5b shows the comparative analysis of the Effective I_Blue_ values for creatinine and interfering molecules, in which no changes in color and signal were seen for all of the interfering molecules compared to the creatinine. This happens because of the absence of creatinine in the measuring solution, which is only responsible for generating a reddish-orange color meditated through PA. Mathematically, the selectivity of the system was determined by calculating the selectivity coefficient (K_Sel_) using Equation (3).

(3) $$K_{Sel}=\frac{{\left(Signal\right)}_{interferent}}{{\left(Signal\right)}_{Cr}}\;$$

where *K_Sel_* is the selectivity coefficient, *(Signal)_interferent_* is the signal value observed when the sensor was checked for the interfering molecules, and *(Signal)_Cr_* is the signal observed for the creatinine molecule, with the addition of picric acid.

It was observed that the k_sel_ for the interfering molecules was <<<1, which indicates that the developed biosensor is highly sensitive and selective towards creatinine. The t-test was also conducted, and the *p*-value was determined for the interfering molecules present in the serum sample against creatinine. The value observed was <0.003 (*n* = 3), which shows that the readings are statistically insignificant. In the serum sample, creatinine is present with the different interfering molecules together; therefore, we have performed a mixed-sample analysis containing all the molecules with creatinine under similar experimental conditions. To evaluate this, the creatinine was mixed with the interfering molecules in the same concentration as in the serum, and the creatinine detection was performed using the developed biosensor. In this case, the sensitivity for the creatinine detection was observed to be 94.8% (*n* = 3) compared to that when the creatinine was tested separately. Thus, the developed sensor was able to selectively detect the creatinine even in the presence of other molecules, i.e., the mixed sample.

### 3.5. Analysis of Spiked Samples

The implication of the fabricated creatinine biosensing device was validated in spiked serum samples because it is considered as a valuable matrix for diagnosing renal dysfunction [56]. The spike-recovery model was employed for this purpose, in which the known creatinine concentrations were added to the serum sample and the signals were compared with the standard calibration plot. The % recovery of creatinine in the spiked serum samples was calculated at different concentrations using Equation (4).

(4) $$\%\;Recovery=\frac{{\left[A\right]}_{Cr}-{\left[B\right]}_{Cr}}{{\left[C\right]}_{Cr}}$$

where **[*A*]*_Cr_* and **[*B*]*_Cr_* are the measurements of creatinine in the spiked and blank samples, respectively; and **[*C*]*_Cr_* is the amount of creatinine present in the standard solutions.

**Figure 6:**
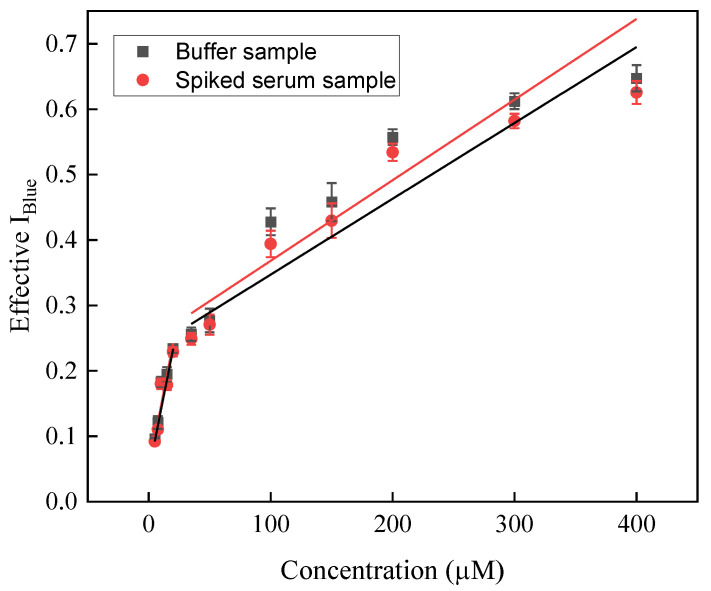
Scatterplot showing the dose-dependent creatinine detection using the sensing probe in standard buffer and spiked serum samples.

Figure 6 represents a concentration-dependent analytical signal of creatinine in a spiked serum sample in which a proportional increase was observed in the Effective I_Blue_ values with the increase in creatinine concentrations. It was also observed that no color change was visible in the sample in which no creatinine was added (data not shown).

Further, to find the sensitivity of the system, the % recovery test was performed in a concentration range of 5–400 µM and it was found that the sensor was able to quantify the creatinine between 89.71 and 97.30% from the spiked serum samples (*n* = 3, RSD < 3.3%). The regression equations for creatinine in the spiked serum samples are represented for low (5–20 µM) and high (35–400 µM) concentrations as follows: Effective I_Blue_ (a.u.) = 0.046 (±0.009) + 0.010 (±0.002) [Cr(µM)] and Effective I_Blue_ (a.u.) =  0.23 (±0.001) + 0.001 (± 0.011) [Cr(µM)] with adjacent R^2^ values of 0.93 and 0.91, respectively. The limit of detection of creatinine in the spiked serum sample was determined to be 32.54 (±1.47) nM, depending upon the standard deviation of three repeated measurements of the blank (95.0% confidence level, *n* = 3). The detailed obtained values are represented in Table 2 showing the recovery % and RSD values. Interestingly, the concentration of creatinine was noted, calculated in the spiked serum sample experiments, and found to be within the clinical range. Such an observation indicates that the developed sensor system could detect creatinine in spiked serum samples.

### 3.6. Device Design

**Figure 7:**
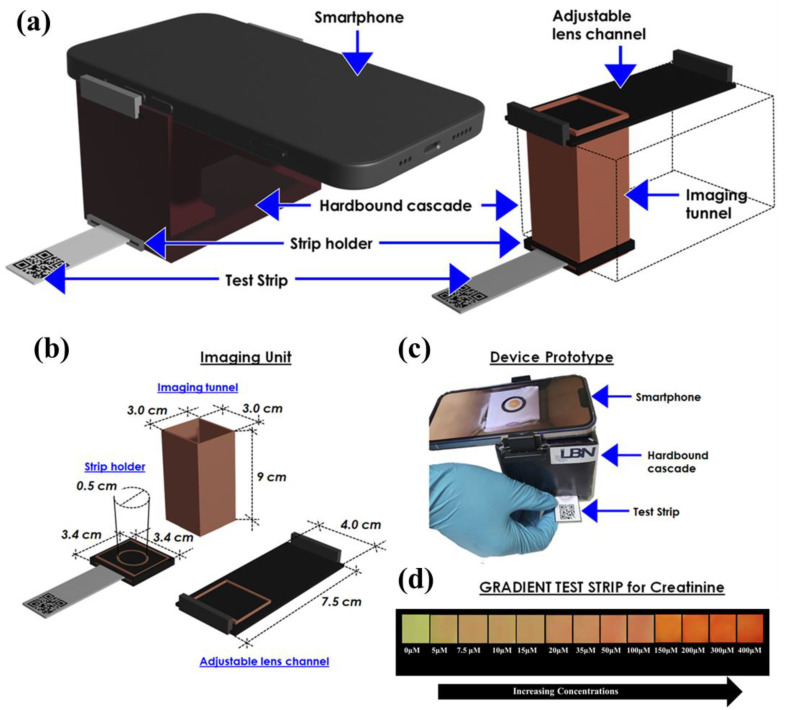
(**a**) Detailed design of the device setup developed for the creatinine measurement; (**b**) Detailed dimension of the designed tunnel and the strip for image capturing and processing; (**c**) Real image of the developed system taking reading for creatinine detection; and (**d**) gradient strip for qualitative detection of creatinine.

The results obtained after all the analyses and testing, including the real samples, proved that the developed device prototype is capable of diagnosing chronic kidney disease in a simplified manner by detecting creatinine in clinical ranges. For translating the device into clinics and for personalized use, the device has been fabricated as follows. The device has been designed using a hard case with dimensions of 8.0 cm (*L*) × 7.5 cm (*W*) × 9.0 cm (*H*), onto which a smartphone is attached, and the test strip was inserted for the detection as shown in Figure 7a. A tunnel-type structure was developed for image acquisition which helps in improving the picture sensitivity so the system is not affected by any atmospheric/interfering light. The dimension of the tunnel structure (3 cm (*L*) × 3 cm (*W*) × 9 cm (*H*)), the strip holder (3.4 cm × 3.4 cm), and the adjustable lens channel are depicted in Figure 7b. This setup assisted in performing the detection and image analysis in one single frame and also helped reduce the need for a controlled laboratory setup, easing the diagnosis process. Additionally, the paper strip showing the reaction zone (diameter = 5.0 mm) was fabricated, onto which the final probe (C/DZ/EDC-NHS/anti-CR) was set up to detect creatinine. This provides a particular spot and allows the reaction to occur in a particular area, which helps in improving the color intensity. A real image of the device performing the detection after the insertion of the test strip is shown in Figure 7c to show the actual device developed. A color base Test Strip has also been developed in which the user can also obtain the broad concentration of creatinine in the sample matrix, as shown Figure 7d.

### 3.7. Storage, Stability and Reproducibility Test

The final sensing probe, i.e., C/DZ/EDC-NHS/anti-CR, was stored at 4 °C in a moist environment, and the probe was tested every week for creatinine detection, and Effective I_Blue_ was recorded. In addition to this, a study for the shelf-life of the designed probe was also performed in which Effective I_Blue_ of the probe was checked every three days. The results of the Effective I_Blue_ values signify that the sensor system preserves a sensitivity of about 96.5% up to three weeks, and the decrease in the signal was recorded up to 90.5 (±1.51)% till the 28th day. The sensing device probe shows an acceptable shelf-life of up to 30 days which is most likely due to the stable immobilization of anti-CR and the retainment of its biological activity during the storage period. It was observed that after four weeks, there was a decrease in the signal, which was up to 85.6%. The most probable reason for this decrease in signal was either the reduction in the biological activity of anti-CR and/or the degradation of the chemical modification steps. The sensor was also tested intraday and interday, and the variation in the signal was found to be 4.1%, indicating the method can be repeated and reused. Additionally, the reproducibility was tested for the biosensing device which showed the RSD was <4.4% and the disc-to-disc RSD was <3.9% even when the fabrication was performed using the same fabrication steps. These slight variations were observed probably because of the negligible variation during the integrated sensor device design phase and some were due to handling errors.

## 4. Conclusions

A paper-based optical sensing device was developed for the point-of-care quantitative monitoring of creatinine in serum samples. The detection was based on a modified chemical reaction to enhance selectivity and sensitivity. As per our knowledge, this is a first-of-its-kind attempt to make a reaction specific for creatinine by fabricating a probe that includes anti-CR antibody on a paper matrix. The change in color was captured, and image processing was performed based on an optical analysis. In this case, the change in the Effective I_Blue_ values was the major factor. It was noted that under optimal conditions, the developed system shows two different dynamic ranges: 5–20 μM and 35–400 μM, including the clinical range of creatinine in humans with a limit of detection of 15.37 (±0.79) nM. Additionally, the developed device shows a highly specific and accurate analytical performance when tested in the spiked serum sample. Further, the probe shows excellent selectivity toward creatinine in the presence of other proteins and ions in the spiked serum sample. The calculated selectivity of the developed sensor was 94.8% (ksel << 1; *p* < 0.003; *n* = 3) and shows a recovery of 89.71–97.30% in spiked serum samples, proving its exceptional potential in the field of medical diagnostics. The developed sensor is promising, comparable, and proven to be an improvement on the already existing optical sensors in terms of its dynamic range, limit of detection, and response time. The most innovative thing of the developed sensor is that it is able to detect creatinine in clinical ranges in which most of the already existing sensors fail either in lower or higher concentrations. Another interesting feature is that we have tried to develop a palm-sized device so that it can easily be implemented in real settings and can also be operated as a personalized diagnostic device. Therefore, in the future this device could possibly be used for the diagnosis of chronic kidney disease with a high specificity and sensitivity.

## Figures and Tables

**Table 1:** Comparison table of some globally developed creatinine sensors with the developed setups in terms of fabrication, response time, analytical performance.

Sr. No.	Analyte	Detection Method	Sensor Configuration	Time	Real Sample	Detection Range	Clinical Range	Point-of-Care Device Prototype	Limit of Detection	Reference
**1.**	Creatinine	Optical (Colorimetric)	CuNPs have been integrated with L-cysteine for selective and sensitive interaction with Creatinine	20 min	Serum and Urine matrix	0.33–5.33 μM	NO	NO	0.454 nM	[45]
**2.**	Creatinine	Optical (Colorimetric)	ABTS was introduced and entrapped in fluorine-doped tin oxide-modified chitosan film	12.5 min	Urine matrix	0–21300 μM	YES	NO	400 μM	[46]
**3.**	Creatinine	Colorimetric	3D-printed element was integrated with smartphone for detection using Hue channel intensity in Jaffe method	6 min	Urine matrix	1000–2000 μM	NO	NO	350 μM	[47]
**4.**	Creatinine	Colorimetric	3,5-dinitrobenzoate was used for quantification by targeting green channel intensity	6 min	Urine matrix	820–10^5^ μM	NO	NO	270 μM	[47]
**5.**	Creatinine	Fluorescence	Glutathione based copper nanoclusters were designed using ascorbic acid for turn-on mode	15 min	Urine matrix	30–1000 μM	YES	NO	13.0 μM	[48]
**6.**	Creatinine	Optical	C-gold nanocomposite was designed using carbon nanodots derived from *Citrullus lanatus* for photoluminescent imaging	10 min	Not Performed	17–1700 μM	YES	NO	6.19 μM	[49]
**7.**	Creatinine	Optical	H_2_O_2_ released through creatinine conversion was detected by reacting it with 4-aminophenazone and hydroxybenzoic acid on a μPAD	15 min	Urine matrix	221–2210 μM	NO	NO	176 μM	[43]
**8.**	Creatinine	Optical (fluorescence)	Gluten stabilized gold quantum cluster was developed to form on/off system for creatinine	3 hr	Serum matrix	20–520 μM	YES	NO	2 nM	[50]
**9.**	Creatinine	Optical	Silver nanoparticles were capped with 2,2-thiodiacetic acid to react with creatinine	5 min	Serum matrix	0.01–1 μM	NO	NO	3 nM	[44]
**10.**	Creatinine	Optical (Colorimetric)	Gold nanoparticles were modified with mercury and detection was performed on synergistic coordination	5 min	Urine matrix	15–35 µM	NO	NO	19.8 nM	[51]
**11.**	Creatinine	Optical (Colorimetric)	Silver nanoparticles capped with citrate for creatinine detection	1 min	Urine matrix	0–4.2 µM	NO	NO	53.4 nM	[52]
**12.**	Creatinine	Optical	3,5 dinitrobenzoic acid was used as chromophore for creatinine detection	2 min	Urine matrix	10–30 µM	NO	NO	Not reported	[53]
**13.**	Creatinine	Optical	Methylamino phenol sulfate was used for oxidation using copper sulfate in presence of creatinine	30 min	Serum matrix	4.4–620 µM	YES	NO	145 nM	[54]
**14.**	Creatinine	Optical	Gold nanoparticles were capped with citrate for the detection of creatinine	24 min	Urine matrix	0.1–20 mM	NO	NO	80 µM	[55]
**15.**	Creatinine	Optical	C/DZ/EDC-NHS/Anti-CR/Cr	7 min	Serum matrix	5–400 μM	YES	YES	15.37 nM	This work

**Table 2:** Recovery table showing percent recoveries of creatinine in spiked serum samples and RSD values.

S.No	Spiked (μM)	Recovered (μM)	Recovery (%)	RSD (%)
1	5	4.77 (±0.05)	95.40	1.10
2	7.5	6.75 (±0.18)	90.09	2.80
3	10	9.54 (±0.25)	95.46	2.66
4	15	13.45 (±0.26)	89.71	1.96
5	20	19.46 (±0.39)	97.30	2.02
6	35	32.99 (±0.99)	94.27	3.02
7	50	47.31 (±1.41)	94.62	2.98
8	100	92.55 (±2.92)	92.55	3.15
9	150	139.99 (±4.61)	93.32	3.29
10	200	189.29 (±5.39)	94.64	2.84
11	300	288.73 (±3.62)	96.24	1.25
12	400	384.50 (±4.01)	96.12	1.04

## Data Availability

Not applicable.
